# Knowledge of journal impact factors among nursing faculty: a cross-sectional study

**DOI:** 10.5195/jmla.2017.207

**Published:** 2017-04

**Authors:** Maha Kumaran, Chau Ha

## Abstract

**Objective:**

The research assessed nursing faculty awareness and knowledge of the journal impact factor (JIF) and its impact on their publication choices.

**Methods:**

A qualitative cross-sectional questionnaire was developed using Fluid Survey and distributed electronically to nursing faculty and instructors at three post-secondary institutions in Saskatchewan. Data were collected on place and status of employment, knowledge and awareness of JIFs, and criteria used to choose journals for publication.

**Results:**

A total of forty-four nursing faculty and instructors completed the questionnaire. The authors found that faculty lack awareness or complete understanding of JIFs and that JIFs are not the most important or only criterion used when they choose a journal for publication.

**Conclusions:**

There are various reasons for choosing a journal for publication. It is important for librarians to understand faculty views of JIFs and their criteria for choosing journals for publication, so that librarians are better equipped to guide researchers in considering their academic goals, needs, and personal values.

## INTRODUCTION

Originally devised in 1955 [1] and first published in its current form in 1972 by Thomson Reuters’s Web of Science, the journal impact factor (JIF) was primarily designed to help librarians decide on journal subscriptions but later evolved into an indicator of journal prestige and quality [2]. The JIF “indicate[s] the number of times, on average, that a citable item in a given journal is…cited over a two-year period in journals indexed by [Journal Citation Reports] JCR” [2] and is the “ratio between citations and recent citable items published” [1]. Nursing journals are rated in both the science and social science editions of the JCR. Although other metrics such as the Eigenfactor score, SCImago journal rank indicator, or journal evaluation tool exist, the JIF is most well-established and oldest citation metric.

Previous literature indicates that academic scholars, including nursing scholars, often feel pressured to publish in journals with a high JIF to receive funding and to be appraised for tenure, promotion, and performance, as these journals are seen as more influential [3–5]. In some countries, JIF policies are used to rank faculty and/or their institutions for recruitment, promotion, and research grant provisions or to bestow monetary compensation on authors who publish in journals with high JIFs [6, 7]. However, there are problems with using the JIF for such purposes [6]. One problem is that the JIF unfairly rates nursing journals as underperforming against journals in other disciplines [8]. As such, it is unfair to compare the JIF across different disciplines, as some disciplines engage in particular research methodologies that are most amenable to the JIF. Olson likens the JIF to nuclear energy, as it can be abused if it falls into the wrong hands [9]. Jackson, Haigh, and Watson scanned nursing journals with the highest JIFs and found them to be more international and general in nature, thus lacking the specialty focus that many authors desire [3].

The JIF has been criticized for how it is measured [10] and what it measures. The JIF emphasizes the “quantity of citations over quality of substance” [11] and can be manipulated by self-citation and publication of review articles. Nursing articles usually focus on clinical and multidisciplinary research, and the emphasis on review articles for impact purposes often puts nursing journals at a disadvantage. Lozano, Larivière, and Gingras have found that the relationship between JIFs and the actual number of citations received by articles is weakening [12]. Polit and Northam listed a plethora of concerns such as self-citation issues and bias toward English-language and North American articles, which could mislead authors into publishing in a journal that is not the best venue for their research [2]. Studies also show that different types of articles have different JIFs. For example, compared with review articles [13, 14], surgical articles [15] and those that are clinically or practically oriented have lower rates of citation [10, 16], thereby misrepresenting the value of the journals they are published in. Despite these criticisms, the number of nursing journals with a JIF has increased from a mere 9 in 1983 [2] to 116 and 114 in the Social Sciences Citation Index and Science Citation Index Expanded, respectively, in 2015.

JIFs should be neither the only measure of journal success nor the primary reason to publish in a specific journal [2, 9, 17]. In a previous study utilizing focus groups, interviews, and a survey [18], health professional and nursing participants identified their top three criteria for choice of publication venue as readership, reputation of the journal, and length of time to publication. Evidence suggests that while “the process and politics of bibliometrics” [19] cannot be avoided, it is important to choose a journal based on its subject, scope, and audience in addition to whether it is peer-reviewed and possibly open access [20]. Nursing scholars are encouraged to understand the “nature of impact” and have dialogues “about the ways in which review processes can account for the many ways the impact of research can be demonstrated” [21]. In this paper, the authors aimed to learn whether nursing faculty in Saskatchewan are aware of the JIF and its meaning and whether this knowledge has any impact on their choices of a journal for publication.

## METHODS

A qualitative cross-sectional questionnaire was designed and developed using Fluid Survey and electronically distributed to nursing faculty and instructors at the University of Saskatchewan, Saskatchewan Polytechnic, and University of Regina, which were the only 3 institutions in the province of Saskatchewan, Canada, that offered various certificate and degree programs in nursing. The survey questionnaire (supplemental appendix) contained 10 open- and closed-ended questions and was open from March 16 to March 31, 2015. Two follow-up emails were sent to the group of initial contacts to increase the response rate. The survey reached a total of 475 nursing faculty and instructors. Sixty-three initiated the survey, and 44 completed the survey, yielding a participation rate of 9%. Of the 44 who completed the survey, 21 were tenured or tenure-track faculty and therefore were required to publish.

## RESULTS

Nursing faculty and instructors were asked whether they knew of the JIF and, if they did, were asked to define the JIF in their own words. Twenty-eight respondents (63%) reported that they knew about the JIF, but their definitions suggest that they did not have a clear understanding of how the JIF is calculated or why only some journals receive a JIF. A few examples of their responses are:

“An impact factors means that the journal is respected by scientific community and highly subscribed”

“ok i thought I knew what this meant, but I looked it up and I didn’t get it right!”

“How many people read the journal. How well known it is for its information”

“It is a rank that is given to a journal in relation to how it is valued by peer reviewers based citations”

When asked if the JIF had any impact on their tenure and promotion, 51% of respondents indicated that they were not sure, 35% said it did not make any difference in their tenure or promotion processes at their institutions, and 14% said it did make a difference. Among tenured and tenure-track faculty, 38% said they were not sure, 33% said it did not make a difference, and 29% said it did make a difference.

**Figure 1:**
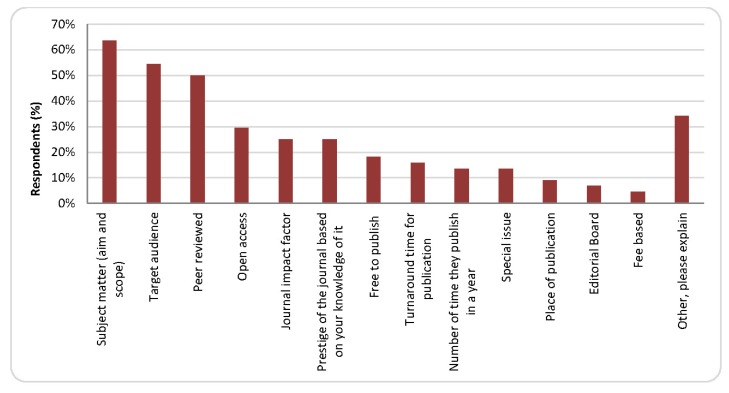
Criteria for choosing a journal for publication among nursing faculty and instructors

Respondents were asked to indicate the criteria they used to choose journals for publication within the last 5 years. The top 3 criteria were (1) the subject matter (aim and scope) of the journal, (2) the target audience, and (3) peer review. Only 25% of respondents choose the JIF as a factor in their choice of a journal for publication (Figure 1). Considering only tenure and tenure-track faculty, the top 3 criteria again were (1) subject matter (95%), (2) target audience (95%), and (3) peer review (76%).

Some respondents associated high JIFs with better dissemination of research and perceived value of journals, both of which they believed to be beneficial to career advancement. Many respondents (50%) indicated that they were interested in learning more about the JIF for themselves, others said they would like to arrange sessions to educate all faculty on the JIF (25%), and a few others (18%) wanted to invite a librarian to their class rooms to talk to students about the JIF.

## DISCUSSION

Our survey results suggest that nursing faculty may not have a clear understanding of the JIF and that the JIF is not a major factor in their choices for publication. Respondents categorized their publication choices into four focus areas: Canadian nursing journals, nursing research journals, nursing education journals, and nursing specialty journals. These publications choices are mainly based on aim, scope, and audience of the journals, and the interdisciplinary nature of nursing research is also taken into consideration, which is consistent with previous literature [7, 17].

Our results increase librarians’ knowledge about faculty awareness and perceptions of the JIF as well as the nature of publication choices and their impact in nursing scholarship. Knowing what JIFs mean to faculty can help librarians better understand, converse with, and educate faculty on the choice of a journal based on individual academic goals, organizational needs, and personal values. This type of consultation may be particularly valuable when faculty apply for grants and scholarships that may require them to publish in high impact journals. Personal goals and values should be taken into consideration during conversations between faculty members and librarians, thus helping librarians to offer unbiased information during the consulting process.

The small number of survey respondents makes it difficult to draw generalized conclusions regarding nursing faculty’s knowledge and perception of the value of the JIF. A follow-up study employing interviews with faculty who are required to publish may yield richer qualitative results. Nonetheless, our findings provide a foundation for further research and discussion on this topic and can help librarians provide unbiased recommendations to faculty and graduate students on publication choices.

## Supplemental File

Appendix Questionnaire

## References

[1] Johnstone MJ (2007). Journal impact factors: implications for the nursing profession. Int Nurs Rev.

[2] Polit DF, Northam S (2011). Impact factors in nursing journals. Nurs Outlook.

[3] Jackson D, Haigh C, Watson R (2009). Editorial: nurses and publications – the impact of the impact factor. J Clinical Nurs.

[4] Katz A (2012). The impact of the impact factor. Oncology Nurs Forum.

[5] Smith KM, Crookes E, Crookes PA (2013). Measuring research ‘impact’ for academic promotion: issues from the literature. J Higher Educ Policy Manag.

[6] Ketefian S, Dai YT, Hanucharurnkul S, Mendes IAC, Norman IJ (2010). Environments for nursing scholarship and journal impact factor in five countries. Int Nurs Rev.

[7] Monastersky R (2005). The number that’s devouring science. Chron Higher Educ.

[8] Dougherty MC, Freda MC, Kearney MH, Baggs JG, Broome M (2011). Online survey of nursing journal peer reviewers: indicators of quality in manuscripts. West J Nurs Res.

[9] Olson CA (2011). Evaluating the quality of a journal: JCEHP’s 2010 impact factor. J Contin Educ Health Prof.

[10] Neuberger J, Counsell C (2002). Impact factors: uses and abuses. Eur J Gastroenterol Hepatol.

[11] Parse RR (2012). Impact factor-one-size-fits-all: what’s wrong with this picture?. Nurs Sci Q.

[12] Lozano GA, Larivière V, Gingras Y (2012). The weakening relationship between the impact factor and papers’ citations in the digital age. J Am Soc Inf Sci Technol.

[13] Oermann MH, Shaw-Kokot J (2013). Addressing the impact factors of nursing education journals. J Nurs Educ.

[14] Watson R, Cleary M, Hunt GE (2013). What gets highly cited in JAN? can editors pick which articles will contribute to a journal’s impact factor?. J Advanced Nurs.

[15] Kodumuri P, Ollivere B, Holley J, Moran CG (2014). The impact factor of a journal is a poor measure of the clinical relevance of its papers. Bone Joint J.

[16] Crookes PA, Reis SL, Jones SC (2010). The development of a ranking tool for refereed journals in which nursing and midwifery researchers publish their work. Nurs Educ Today.

[17] Hunt GE, Happell B, Chan SWC, Cleary M (2012). Citation analysis of mental health nursing journals: how should we rank thee?. Int J Mental Health Nurs.

[18] Regazzi JJ, Aytac S (2008). Author perceptions of journal quality. Learned Publ.

[19] Davidson PM, Newton PJ, Ferguson C, Daly J, Elliott D, Homer C, Duffield C, Jackson D (2014). Rating and ranking the role of bibliometrics and webometrics in nursing and midwifery. Sci World J.

[20] Watson R, Cleary M, Jackson D, Hunt GE (2012). Open access and online publishing: a new frontier in nursing?. J Advanced Nurs.

[21] Ironside PM (2007). Advancing the science of nursing education: rethinking the meaning and significance of impact factors. J Nurs Educ.

