# Supplementary material for: Knowledge of journal impact factors among nursing faculty: a cross-sectional study
Source: J Med Libr Assoc. 2017 Apr;105(2):140–4. doi: 10.5195/jmla.2017.207 (PMC5370604; doi:10.5195/jmla.2017.207)
Supplement: Appendix [file jmla_apr17_kumaran_app.pdf]

JMLA Journal of the Medical Library Association

SC

Supplemental content to
J Med Libr Assoc. Apr;105(2): dx.doi.org/10.5195/jmla.2017.207
www.mlanet.org
© Kumaran, Ha 2017

Knowledge of journal impact factors among nursing faculty: a cross-sectional study

Maha Kumaran MA, MLIS; Chau Ha, MLIS, BscN

APPENDIX

Questionnaire

1. Where are you employed? (Check one)
   - o University of Saskatchewan
   - o University of Regina
   - o Saskatchewan Polytechnic
2. What is your employment status with the institution? (Check all that apply)
   - o Tenured
   - o Tenure track (regular, academic programming appointment)
   - o Sessional
   - o Full time (teaching, clinical, and labs)
   - o Part time (teaching, clinical, and labs)
   - o Other, please specify
3. What is the length of your employment within the organization you teach? Please respond in number of years or months (example: 3 months or 2 years) (Free text)
4. Do you know about journal impact factors? (Check one)
   - o Yes
   - o No
5. If yes to question 5, (Check one)
   - o Define journal impact factors in your own words
   - o Is the journal impact factor important in your decision as to whether or not to publish in a particular journal? Please explain.
6. Name titles of journals that you have published within the last 5 years. If you haven't published in the last 5 years or if you are not expected to publish, please indicate this. (Free text)
7. What criteria did you use to decide to publish with the above journals? (Check all that apply)
   - o Target audience
   - o Subject matter (aim and scope)
   - o Journal impact factor
   - o Place of publication
   - o Prestige of the journal based on your knowledge of it
   - o Editorial board
   - o Turnaround time for publication
   - o Number of times they publish in a year
   - o Fee based
   - o Free to publish
   - o Open access

SC
Supplemental content to
J Med Libr Assoc. Apr;105(2): dx.doi.org/10.5195/jmla.2017.207
www.mlanet.org
© Kumaran, Ha 2017

- o Peer reviewed
- o Special issue
- o Other, please explain

8. Impact factor and tenure (Check one)
   - o Journal impact factor makes a difference during my tenure and/or promotion process at my institution
   - o Journal impact factor does not make any difference during my tenure and/or promotion process at my institution
   - o I am not sure if journal impact factor makes a difference during my tenure and/or promotion process at my institution
9. Help learning about journal impact factors. (Check all that apply)
   - o I personally would like to learn more about journal impact factors and how they can help me
   - o I would like to invite the librarian to my class to talk about journal impact factors
   - o I am not interested in journal impact factors as this doesn't apply to me
   - o I already know all there is to know about journal impact factors
   - o I would like to arrange a session so all faculty can learn about journal impact factors
10. What are your perceptions about the impact of publishing in a high impact factor journal for research or knowledge sharing within your discipline? (Free text)
